# Accurate descriptions of molecule-surface interactions in electrocatalytic CO_2_ reduction on the copper surfaces

**DOI:** 10.1038/s41467-023-36695-7

**Published:** 2023-02-20

**Authors:** Zheng Chen, Zhangyun Liu, Xin Xu

**Affiliations:** 1 Collaborative Innovation Center of Chemistry for Energy Materials, Shanghai Key Laboratory of Molecular Catalysis and Innovative Materials, MOE Key Laboratory of Computational Physical Sciences, Department of Chemistry, Fudan University, 200433 Shanghai, People’s Republic of China; 2 Hefei National Laboratory, 230088 Hefei, P. R. China

**Keywords:** Heterogeneous catalysis, Density functional theory, Materials for energy and catalysis

## Abstract

Copper-based catalysts play a pivotal role in many industrial processes and hold a great promise for electrocatalytic CO_2_ reduction reaction into valuable chemicals and fuels. Towards the rational design of catalysts, the growing demand on theoretical study is seriously at odds with the low accuracy of the most widely used functionals of generalized gradient approximation. Here, we present results using a hybrid scheme that combines the doubly hybrid XYG3 functional and the periodic generalized gradient approximation, whose accuracy is validated against an experimental set on copper surfaces. A near chemical accuracy is established for this set, which, in turn, leads to a substantial improvement for the calculated equilibrium and onset potentials as against the experimental values for CO_2_ reduction to CO on Cu(111) and Cu(100) electrodes. We anticipate that the easy use of the hybrid scheme will boost the predictive power for accurate descriptions of molecule-surface interactions in heterogeneous catalysis.

## Abstract (web summary)

The most widely used density functional approximations for heterogenous catalysis has limited accuracy. Here, the authors propose a hybrid scheme to accurately describe Cu surface for CO_2_ electroreduction, facilitating the rational design of catalysts.

## Introduction

Copper is a major component in several important industrial catalysts, such as those for the water–gas shift reaction^1^, and methanol synthesis from the synthesis gas mixture^2–4^, etc. It has recently emerged as a key ingredient in some high-potential electrocatalysts for the CO_2_ reduction reaction (CO_2_RR) to produce valuable fuels and chemicals^5–9^. In building up these processes, the first-principles calculations play a key role in understanding the mechanisms and achieving the catalyst’s rational design^5,9–12^.

Density functional theory (DFT) has been the method of choice for quantitative understanding and developing of complex systems in either quantum chemistry or computational materials science. Often, hybrid functionals are widely used for molecules and solids with localized electrons, while generalized-gradient approximations (GGAs) usually suffice for bulk and surface metals with delocalized electrons^13–15^. However, the choice is not trivial when dealing with systems as in heterogeneous catalysis where molecules meet metal surfaces, both of which ought to be simulated accurately.

**Fig. 1:**
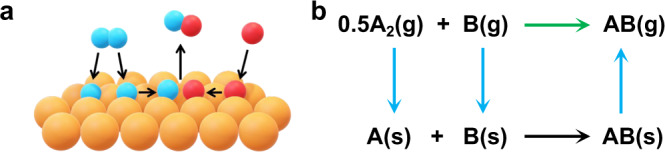
Accurate description for heterogeneous catalysis. Accurate descriptions of both the molecular and the extended system are essential for an accurate description of the molecule–metal-surface interactions in heterogeneous catalysis. **a** The feature of metal-surface catalysis. The targets are to break some old bonds and to form some new bonds in the gas-phase molecular system (blue and red balls), while the metal surface (orange balls) assists such processes by stabilizing the intermediate molecular fragments (blue and red balls). **b** A Born–Haber cycle invoked in metal-surface catalysis. The gas-phase reaction (green) and the surface reaction (black) are related by the processes of adsorption and desorption (blue).

For metal-surface catalysis, the targets are to break some old bonds in the reactant molecules and to form some new bonds in the product molecules, where the metal surface temporarily stabilizes the intermediate molecular fragments, thus catalyzing the whole process (Fig. 1a). By employing a Born–Haber cycle (Fig. 1b), the gas-phase reaction (green) and the surface reaction (black) are connected by the processes of adsorption and desorption (blue). The formation energy of a surface species, which quantifies both the internal stability of the molecular fragment and the external stability due to the presence of the metal surface, constitutes the free energy profile and determines the catalytic kinetics on the surface^11,16–18^. The formation energy of a surface species can also be decomposed into two contributions. One is the formation energy of the corresponding molecular fragment in the gas phase, while the other is the adsorption energy of this molecular fragment to the extended surface. This illustrates that accurate descriptions of both the molecular and the extended systems are essential for an accurate description of metal-surface catalysis.

However, most theoretical approaches to study metal-surface catalysis are based on GGAs, which are known to perform poorly in the prediction of reaction energies and reaction barriers in the gas phase^19–22^. Such errors in the gas phase will be carried over to the description of the surface processes, although some errors will be luckily canceled out to some extent in certain circumstances. It is well-known that some widely used GGAs predict incorrectly the preferred CO adsorption sites on many metal surfaces^23^, whereas the adsorbed CO, denoted as *CO where * indicates a surface site, is a key intermediate involved in many catalytic processes. Grand effort has been devoted to improving the accuracy in the calculations of metal-surface catalysis. Some semiempirical methods^22^ have been proposed at the low-rung levels of Jacob’s ladder for density functional approximations (DFAs). For instance, the specific reaction parameter (SRP) approach has achieved chemically accurate descriptions of sticking parameters in many molecule–metal-surface systems^24,25^, while a semiempirical method to correct the GGAs-based errors in the gas-phase reactions shows a substantial improvement of the calculated equilibrium and onset potentials for CO_2_RR to CO on Au, Ag, and Cu electrodes^22^. Besides, the random phase approximation (RPA) standing on the top rung of the Jacob’s ladder for DFAs has been utilized for accurate descriptions of metal-surface catalysis^26–28^. The embedded high-level correlated wavefunction methods have also been employed for describing both the ground state and the excited state metal surface reactions^9,29,30^. Noteworthily, it has also been shown that the doubly hybrid approximations, e.g., XYG3 and XYGJ-OS^19,31,32^, can achieve good accuracy for molecules^19,20^, the extended semiconductors and insulators^21^, as well as metal-ligand bondings^33^, while their performance for describing molecule–metal-surface interactions still await to be explored.

Here, we apply a hybrid scheme, XYG3:GGA, that combines the XYG3 functional^19,32^ and the periodic GGA, to describe some key steps in the copper-based heterogeneous catalysis. The accuracy of XYG3:GGA is validated by a benchmark set, where accurate experimental results are available, which includes (1) the preferred CO adsorption sites on Cu(111) and Cu(100) surfaces, (2) the adsorption energies of CO, H, and O on the Cu(111) surface, and that of NH_3_ on the Cu(100) surface, (3) the H_2_ dissociation barrier and the 2 *H desorption barriers on the Cu(111) surface. The benchmark results show that the XYG3:GGA scheme provides a prediction close to chemical accuracy for all these well-established cases. Finally, we utilize the XYG3:GGA scheme to study the electrocatalytic CO_2_RR to CO on Cu(111) and Cu(100) surfaces. A substantial improvement on the calculated equilibrium and onset potentials is achieved. Taken together, we conclude that the very high accuracy of the XYG3:GGA scheme, as well as its easy use, will enhance the predictive power of the computational catalysis for the copper-based catalysts, which shall offer new mechanistic insights and help catalysts rational design in a quantitative way.

## Results and discussion

**Fig. 2:**
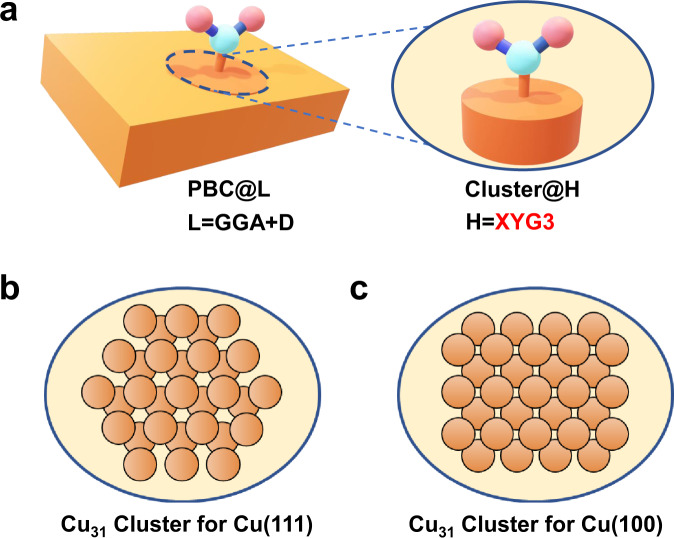
Hierarchy of models and methods for adsorption energy calculations. **a** The delocalization effects of the extended metal surface are taken into account by an efficient periodic boundary condition (PBC) calculation as the basic low level (PBC@L) at the GGA level with the dispersion correction (L = GGA + D), while the calculations on the metal-adsorbate interactions are then updated to the target high level by embedding the clusters (Cluster@H) into their periodic environment. In particular, the double- hybrid functional XYG3 is employed as the target high-level method. The embedded clusters for systems related to adsorptions on Cu(111) and Cu(100) surfaces are chosen as Cu_31_ (**b**) and Cu_31_ (**c**) of specific shapes, respectively. The orange balls represent the Cu atoms.

To obtain the reaction energy and the reaction barrier of a surface elementary reaction (Fig. 1b), we calculate the formation energy $\varDelta {E}_{{{{{{{\rm{S}}}}}}}^{\ast }}^{{{{{{\rm{f}}}}}}}$ of surface species S* by

1 $$\varDelta {E}_{{{{{{{\rm{S}}}}}}}^{\ast }}^{{{{{{\rm{f}}}}}}} = \varDelta {E}_{{{{{{\rm{S}}}}}}({{{{{\rm{g}}}}}})}^{{{{{{\rm{f}}}}}}}+\varDelta {E}_{{{{{{{\rm{S}}}}}}}^{\ast }}^{{{{{{\rm{ad}}}}}}},$$

which applies to either an intermediate or a transition state (TS). The formation energy of a surface species in the gas phase, i.e., $\varDelta {E}_{{{{{{\rm{S}}}}}}({{{{{\rm{g}}}}}})}^{{{{{{\rm{f}}}}}}}$, can be calculated easily at a high-level method, such as XYG3, or even the gold standard CCSD(T), i.e., coupled cluster with single–double and perturbative triple substitutions. The key is how to calculate the adsorption energy $\varDelta {E}_{{{{{{{\rm{S}}}}}}}^{\ast }}^{{{{{{\rm{ad}}}}}}}$ accurately, which is realized by means of the method called XO-PBC^34^, as illustrated in Fig. 2.

XO^35^ is an extension of the well-established “our own n-layered integrated molecular orbital method” (ONIOM^36^) by further allowing the overlapping via a fragmentation scheme, where computation algorithms for energies, gradients, and Hessians are all available. The recent XO-PBC method^34^ goes beyond the standard XO by applying the periodic boundary condition (PBC) in order to deal with extended systems. Here, it is convenient to first carry out an efficient PBC calculation as the basic low level (PBC@L), taking into account the delocalization effects of the extended metal surface, which is then updated to the target high level by embedding the cluster calculation (Cluster@H) into the periodic environment (Fig. 2a). XO in its simplest version used here is comparable to many other embedding approaches^28,36–40^.

According to the XO-PBC scheme^34^, the final total energy is then given by

2 $${E}_{{{{{{\rm{XO}}}}}}-{{{{{\rm{PBC}}}}}}({{{{{\rm{H}}}}}}:{{{{{\rm{L}}}}}})} = {E}_{{{{{{\rm{PBC}}}}}}@{{{{{\rm{L}}}}}}}+({E}_{{{{{{\rm{Cluster}}}}}}@{{{{{\rm{H}}}}}}}-{E}_{{{{{{\rm{Cluster}}}}}}@{{{{{\rm{L}}}}}}}).$$

Thus, the adsorption energy Δ*E*^*ad*^ is calculated as

3 $$\varDelta {E}_{{{{{{\rm{XO}}}}}}-{{{{{\rm{PBC}}}}}}({{{{{\rm{H}}}}}}:{{{{{\rm{L}}}}}})}^{{{{{{\rm{ad}}}}}}} = \varDelta {E}_{{{{{{\rm{PBC}}}}}}@{{{{{\rm{L}}}}}}}^{{{{{{\rm{ad}}}}}}}+(\varDelta {E}_{{{{{{\rm{Cluster}}}}}}@{{{{{\rm{H}}}}}}}^{{{{{{\rm{ad}}}}}}}-\varDelta {E}_{{{{{{\rm{Cluster}}}}}}@{{{{{\rm{L}}}}}}}^{{{{{{\rm{ad}}}}}}}).$$

In particular, we adopt the PBE functional^41^ as the low level, where dispersion contributions are accounted for by using the GGA + D method with the Becke–Jonson (BJ^42,43^) damping. Meanwhile, the XYG3 functional is used as the target high level (more computational details can be found in the Supplementary Methods).

In principle, when the cluster is sufficiently large, the finite cluster effect will become sufficiently small, and the adsorption energy given by Eq. (3) will be sufficiently accurate^34,37^. However, there is an additional issue in regard to the basis set used. To make practical use of Eq. (3), we are looking for a computational scheme that is both accurate and efficient. In molecular science, the high-quality results for CCSD(T) at the complete basis set (CBS) are often approached by a CBS extrapolation from a large basis (LB) set at a low level of method (e.g., the second-order Møller–Plesset perturbation theory, MP2) augmented with the CCSD(T) calculation at a small basis (SB) set^44^. In fact, the electronic state of the metal surface tends to be localized due to the formation of the chemisorption bonds. In this context, we may use a similar strategy to approach the target result at the level of H/LB as

4 $$\varDelta {E}_{{{{{{\rm{H}}}}}}/{{{{{\rm{LB}}}}}}}\,\approx \,\varDelta {E}_{{{{{{\rm{L}}}}}}/{{{{{\rm{LB}}}}}}}+(\varDelta {E}_{{{{{{\rm{H}}}}}}/{{{{{\rm{SB}}}}}}}-\varDelta {E}_{{{{{{\rm{L}}}}}}/{{{{{\rm{SB}}}}}}}).$$

Equation (4) is first validated here for XYG3 by a set of gas-phase reactions, which are the net reactions that have been sedulously pursued in the heterogeneous catalysis (Supplementary Table 1). The results shown in Supplementary Fig. 1 clearly demonstrated that XYG3/def2-QZVP (LB) is able to predict the experimental values for reaction energies accurately, while the predictions of PBE/def2-QZVP are of poor quality (Supplementary Fig. 1a), demonstrating the infamous errors of the GGA functional for gas- phase reactions. The results of XYG3 are basis set sensitive, such that XYG3/def2-SVP (SB) show significantly large errors (Supplementary Fig. 1b). By employing Eq. (4) to combine the values of PBE/def2-QZVP, XYG3/def2-SVP, and PBE/def2-SVP, the results of XYG3/def2-QZVP can be accurately and efficiently reproduced, which can be compared favorably to the experimental values (Supplementary Fig. 1c, d).

By applying Eq. (4) to Eq. (3), the adsorption energy is now calculated by

5 $$\varDelta {E}_{{{{{{\rm{XYG3}}}}}}:{{{{{\rm{GGA}}}}}}}^{{{{{{\rm{ad}}}}}}} =	 \varDelta {E}_{{{{{{\rm{PBC}}}}}}@{{{{{\rm{GGA}}}}}}/{{{{{\rm{PAW}}}}}}}^{{{{{{\rm{ad}}}}}}}+(\varDelta {E}_{{{{{{\rm{Cluster}}}}}}@{{{{{\rm{XYG3}}}}}}/{{{{{\rm{def2}}}}}}-{{{{{\rm{SVP}}}}}}}^{{{{{{\rm{ad}}}}}}}\\ 	-\varDelta {E}_{{{{{{\rm{Cluster}}}}}}@{{{{{\rm{GGA}}}}}}/{{{{{\rm{def2}}}}}}-{{{{{\rm{SVP}}}}}}}^{{{{{{\rm{ad}}}}}}}),$$

where the basis sets are specified. The PBC@GGA calculation is carried out by the projector augmented-wave (PAW) basis with a high kinetic energy cutoff (see Supplementary Methods for details), which is known to well represent the LB^45^. Here, the energy difference between XYG3 and GGA for cluster model calculations are effectively carried out by using the SB of def2-SVP, which enables to efficiently simulate metal clusters with sufficiently large size as Cu_31_ (Fig. 2b) and Cu_31_ (Fig. 2c) for Cu(111) and Cu(100) surfaces, respectively. It has been demonstrated before that the energy difference between H and L converge well with cluster size of appropriate shapes^37^. Here, we refer to Supplementary Fig. 2 for illustrative testing on the cluster size effects for CO adsorption on Cu(111) and Cu(100) surfaces. It is worthy of note that the convergence of the cluster size effect for different metals is not necessarily the same (see Supplementary Fig. 3 for CO adsorption on Au(111) as a comparison). Inspection of the cluster size effect is important for achieving reliable results with the hybrid scheme.

In the following, the accuracy of the current XYG3:GGA scheme by using Eq. (5) will be demonstrated by several benchmark cases, where accurate experimental results are available as summarized in Supplementary Table 2. Besides those for GGAs of PBE and PBE-D3BJ, the performances of several widely used DFAs (i.e., M06-L^23^, B3LYP, B3LYP-D3BJ) are also examined, representing the effects to update the GGAs to meta-GGA, and hybrid GGAs to doubly hybrid XYG3 as the high-level method ascending the Jacob’s ladder.

### Preferred CO adsorption sites

Even though CO interactions with Cu surfaces are of particular interest, there exists a large gap between the experimental observation and the theoretical prediction. While the experiment observed that CO preferred the top site on the Cu(111) surface, previous theoretical calculations showed various possibilities, depending critically on the methods used^23,26,37–39,46^.

Most GGAs predicted a strong preference of the hollow site^23,26,37–39^, while meta-GGAs predicted a weak preference of the hollow site^23^. Hybrid functionals predicted either a weak^38,39^ or a strong^37^ top site preference depending on the functional used, while RPA^26,39^ and an embedded configuration interaction (ECI) theory^46^ predicted a strong top site preference. Thus, it seems that the theoretical results are in better agreement with the experimental result, as the functionals ascend Jacob’s ladder. Furthermore, it was emphasized that RPA and ECI may differ qualitatively, as ECI predicted a positive adsorption energy on the hollow site^46^, which was believed to be inconsistent with the experimental observation where CO was found to adsorb on the hollow site at a high CO coverage^39^. Hence, in addition to the absolute adsorption energy on the top site, the adsorption energy difference between the top and the hollow sites is also an important metric to benchmark the computational methods.

**Fig. 3:**
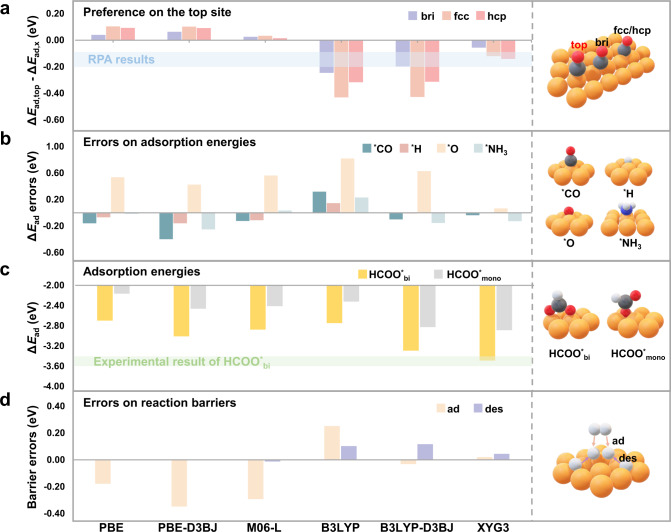
The benchmark set. **a** Preferred CO adsorption site on the Cu(111) surface. A negative value suggests the top site be preferred in accordance with the experiment. The RPA results suggest a top site preference of 0.10 eV^26^ or 0.22 eV^39^ (the horizontal bar in light blue), which is used as an indicator for the adsorption energy difference between the top and the hollow sites. **b** The computation errors for adsorption energies of CO, H, and O on the Cu(111) surface, and NH_3_ on the Cu(100) surface. The experimental results are used as references. **c** The computed adsorption energies for bidentate (HCOO^*^_bi_) and monodentate (HCOO^*^_mono_) formate on the Cu(111) surface. The experimental result of HCOO^*^_bi_ is presented as the bar in light green. **d** The computation errors for barriers of the H_2_ dissociative adsorption and 2 *H desorption on the Cu(111) surface. The experimental results were used as references. Direct periodic PBE and PBE-D3BJ calculations were carried out, while the hybrid scheme was applied to the other functionals for efficiency. The details of the experimental reference data can be found in Supplementary Table 2, while the calculated data are summarized in Supplementary Tables 3–5. The white, gray, red, blue, and orange balls represent H, C, O, N, and Cu atoms, respectively.

Figure 3a presents the CO adsorption energy differences for Δ*E*_ad,top_ – Δ*E*_ad,bri_, Δ*E*_ad,top_ – Δ*E*_ad,fcc_, and Δ*E*_ad,top_ – Δ*E*_ad,hcp_, which indicate the CO adsorption preference on the top site over the other sites (i.e., the bri site, and the fcc or hcp hollow site, bri = bridge, fcc = face-centered cubic, hcp = hexagonal close packed) using different functionals. As seen from the calculated data in Supplementary Table 3, the present M06-L:PBE-D3BJ scheme yields very similar adsorption energies as compared to those from the previous periodic M06-L calculations^23^, where the corresponding adsorption energies are (−0.71 eV vs −0.65 eV^23^) on the top site and (−0.75 eV vs −0.70 eV^23^) on the fcc hollow site, respectively. This good agreement demonstrates the reliability and usefulness of the present hybrid scheme, which is more cost-effective than the direct PBC calculation using higher rung functionals^34^. In consistency with the previous theoretical works^23,26,37–39^, our results also show that GGAs predict a strong hollow site preference (i.e., with a positive energy difference of Δ*E*_ad,top_ – Δ*E*_ad,fcc_ ≈ 0.1 eV), while meta-GGA predicts a weak hollow site preference (Δ*E*_ad,top_ – Δ*E*_ad,fcc_ ≈ 0.04 eV).

We see a hierarchy of improved performance such that hybrid and doubly hybrid functionals predict a strong top site preference (i.e., with a negative energy difference). Nevertheless, B3LYP predicts a positive adsorption energy on the fcc site (Supplementary Table 3), which was considered unreasonable^39^. B3LYP-D3BJ improves the prediction on the absolute adsorption energies, whereas it may still have predicted a too large difference between the top and the hollow sites (Δ*E*_ad,top_ – Δ*E*_ad,fcc_ ≈ −0.43 eV). Noteworthily, XYG3 predicts that the adsorption energy on the fcc site is 0.12 eV weaker than that on the top site, which can be compared favorably to the RPA results of 0.10 eV^26^ or 0.22 eV^39^ (see the horizontal bar in light blue in Fig. 3a).

The common model describing CO adsorption invokes interactions of the two CO frontier orbitals, the highest occupied molecular orbital (HOMO) 5σ and the lowest unoccupied molecular orbital (LUMO) 2π*, with the metal states^47^. It is well-known that GGAs over-stabilize 2π* and de-stabilize 5σ (Supplementary Table 4), due to the infamous delocalization error^48^ for violating the Perdew–Parr–Levy–Balduz (PPLB) linearity condition^49^ (Supplementary Fig. 4). While 2π* prefers the metal-surface bonding in the hollow/bridge sites, 5σ prefers the metal-surface bonding in the top site. Conceivably, it is because that the former wins over the latter, resulting in an erroneous site preference by the low-rung functionals^50^. While Supplementary Fig. 4 demonstrates that the PPLB linearity condition is largely fulfilled by XYG3, such that the 5σ and 2π* orbital levels are nicely reproduced (Supplementary Table 4). XYG3 diminishes the notorious delocalization error, leading to a general improvement as also shown below.

### Adsorption energies

We now pay more attention to the performance of the XYG3:GGA scheme and some other methods in describing the absolute adsorption energies (Supplementary Table 5). It is worth noting that the benchmark values should consider the vibrational zero-point energy (ZPE) contribution contained in the low-temperature experimental surface reaction energy^51^. This approach has also been employed here to consider the ZPE contributions to all the experimental values (see Supplementary Methods 1.2 and Supplementary Table 2 for details). The performances of some selected DFAs on calculating the adsorption energies of CO, H, and O on the Cu(111) surface, as well as the NH_3_ adsorption energy on the Cu(100) surface, are tested, while the calculation errors of different functionals are presented in Fig. 3b.

For CO adsorption on the Cu(111) surface, we compare the calculated values for the on-top molecular adsorption with the experimental value (Supplementary Table 5). The PBE-predicted value is −0.75 eV, which indicates a too-strong adsorption as compared to the experimental value of −0.59 eV^51^. Adding the D3BJ dispersion correction to PBE leads to an adsorption energy of −0.99 eV, further departing from the reference. M06-L performs reasonably well, yielding adsorption energy of −0.71 eV. While B3LYP-predicted CO adsorption is too weak (−0.27 eV), its dispersion correction with D3BJ helps considerably (−0.69 eV). Among the selected functionals, XYG3 gives the best performance, predicting adsorption energy of −0.63 eV, which is closer to the experimental value of −0.59 eV^51^ than are the RPA results of −0.37 eV^39^ and −0.42 eV^26^, respectively.

Supplementary Table 5 also summarizes the other results for NH_3_ molecular adsorption and H_2_ and O_2_ dissociative adsorption. The results clearly demonstrate the excellent performance of XYG3 in describing the adsorption behaviors on the copper surfaces. The mean absolute derivation (MAD) for XYG3 is 0.06 eV, which is close to the chemical accuracy of 0.04 eV. On the other hand, we do not, in general, find a hierarchy of improvement from GGA (e.g., PBE) to meta-GGA (e.g., M06-L) to hybrid-GGA (e.g., B3LYP), and from without the dispersion correction to with the dispersion correction (e.g., PBE vs PBE-D3BJ and B3LYP vs B3LYP-D3BJ) for data in Supplementary Table 5. The poor predictions for the HOMO/LUMO of the adsorbate molecules are general for the commonly used DFT methods, while these frontier orbitals play an important role in forming the adsorption bonds. The delocalization error can be particularly severe due to the high electronegativity of O, which, in turn, leads to a high degree of charge transfer during O adsorption. This provides a plausible explanation why errors for O adsorption energies on the metal surfaces are so large for other methods that suffer from the delocalization error.

Very recently, the experimental adsorption energy of the HCOO· radical bonded to the clean Cu(111) surface has been reported^52^, which provides an important benchmark for validating the accuracy of computational methods (Fig. 3c). The results show that only XYG3 can accurately predict the experimental adsorption energy of the bidentate formate, while most other functionals show very large errors. Considering the good performance on describing the bidentate formate, we suggest that the adsorption energy of monodentate formate predicted by XYG3 could be used as a useful reference, which is difficult to be determined by the experiment. Since both bidentate and monodentate formates are key intermediates in many catalytic processes, such as water–gas shift reaction^1,53^ and methanol synthesis^2–4^, the errors as large as 0.43–0.74 eV for GGAs could lead to a high risk of misunderstanding for the mechanisms.

### Hydrogen activation and desorption barriers

We also test the performance of the XYG3:GGA scheme in describing the kinetic barriers by employing the hydrogen dissociative adsorption and the corresponding desorption on the Cu(111) surface (Fig. 3d). By using the SRP approach, the experimental data for hydrogen dissociative adsorption probability on the Cu(111) surface has been well reproduced with chemical accuracy^25^, leading to a 0.63 eV barrier for the H_2_ dissociative adsorption on the bridge site. This value is considered as an indirect experimental value and is used as the reference here. On the other hand, the 2 *H desorption barrier determined by the temperature-programmed desorption (TPD) is 0.77 eV^54^, which corresponds to 0.84 eV after applying the ZPE contribution correction (Supplementary Table 2).

The errors for the predicted barriers with different functionals are presented in Fig. 3d. While PBE predicts a good desorption barrier, it significantly underestimates the dissociative adsorption barrier. Both PBE-D3BJ and M06-L follow the same trend as PBE, further exacerbating the problem. On the contrary, B3LYP overestimates the dissociative adsorption barrier. Such a tendency is eliminated by adding the dispersion correction as in B3LYP-D3BJ. B3LYP also overestimates the 2 *H desorption barrier to some extent, while B3LYP-D3BJ does not help in this context. Encouragingly, XYG3 can correctly predict both barriers for the H_2_ dissociative adsorption and the 2 *H desorption, which represents an important advance.

Interestingly, an advanced embedded multireference second-order perturbation theory (emb-MRPT2) method has also been applied to the H_2_/Cu(111) system, where a 0.05 eV dissociative adsorption barrier was reported and a 1.00 eV desorption barrier was obtained^55^. These numbers differ significantly from our reference data of 0.63 eV for the former and 0.84 eV for the latter. We tend to believe that the emb-MRPT2 barriers are less reliable, as they indicate dissociative adsorption energy of −0.95 eV for H_2_ on Cu(111). This overshoots the experimental H_2_ dissociative adsorption energies of −0.75 eV and −0.93 eV on Pt(111) and Pd(111) surfaces, respectively^51^, while it is well-established that H_2_ dissociation on Cu is much more difficult than that on Pt, Pd^25,56,57^, indicating that the dissociative adsorption energy for H_2_ on the former should be smaller in magnitude than those on the latter.

### Electrocatalytic CO_2_ reduction on Cu(111) and Cu(100) surfaces

The overall reaction of CO_2_RR to CO can be expressed as

6 $${{{{{{\rm{CO}}}}}}}_{2}({{{{{\rm{g}}}}}})+2({{{{{{\rm{H}}}}}}}^{+}+{{{{{{\rm{e}}}}}}}^{-})({{{{{\rm{aq}}}}}})\to {{{{{\rm{CO}}}}}}({{{{{\rm{g}}}}}})+{{{{{{\rm{H}}}}}}}_{2}{{{{{\rm{O}}}}}}({{{{{\rm{aq}}}}}}).$$

By employing the computational hydrogen electrode (CHE) method^7,58^ (see Supplementary Methods 1.3 for details), the chemical potential of 2(H^+^ + e^-^) at pH = 0 and *U* = 0 V (*U* stands for the electrode potential) is equal to the chemical potential of H_2_ under the standard condition. Here, the XYG3, PBE-D3BJ, and PBE functionals are employed, leading to the reaction free energy of Eq. (6) from CO_2_(g) to CO(g) calculated as 0.32 eV, 0.63 eV, and 0.63 eV, respectively. These results suggest that the predicted equilibrium potentials *U*_eq_ = −Δ*G*_r_/2e^−^ are −0.16 V, −0.32 V, and −0.32 V, respectively. Compared to the experimental *U*_eq_ of −0.10 V^5^, it is clearly that the XYG3 number for *U*_eq_ is quite good, while PBE-D3BJ and PBE predict *U*_eq_ with an obvious derivation.

**Fig. 4:**
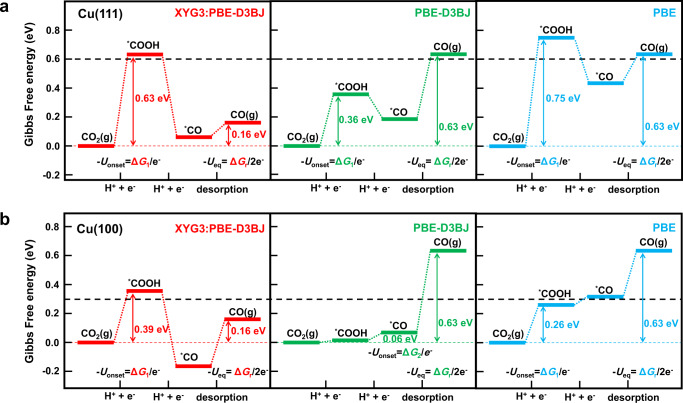
Applications in electrocatalytic CO_2_ reduction to CO on the copper surfaces. Free energy diagrams for CO_2_RR to CO on **a** Cu(111) and **b** Cu(100) electrodes. The corresponding free energy diagrams predicted by XYG3:PBE-D3BJ (red), PBE-D3BJ (green) and PBE (blue) are compared in the context of the computational hydrogen electrode (CHE) method. The black dashed lines at **a** 0.60 eV and **b** 0.30 eV mark the free energies corresponding to the experimental onset potentials *U*_onset_ of Cu(111) (−0.60 V vs the relative hydrogen electrode (RHE)) and Cu(100) (−0.30 V vs RHE), respectively, with which the computed Δ*G*_1_ shall be compared, while the experimental equilibrium potential *U*_eq_ is −0.10 V vs RHE, with which the computed Δ*G*_r_ shall be compared.

As shown in Fig. 1b, the XO framework provides a way to combine the best of different worlds. Thus, the gas-phase reaction (green) may be described by the gold standard CCSD(T), the surface reaction (black) may be described by a periodic method such as PBE-D3BJ, while the processes of adsorption and desorption (blue) may be described by the hybrid XYG3:GGA scheme. Figure 4, denoted as XYG3:PBE-D3BJ, actually depicts such results for CO_2_RR on Cu(111) and Cu(100) surfaces, where the gas-phase reaction is described with CCSD(T). The reaction free energy of Eq. (6) from CO_2_(g) to CO(g) is now calculated as 0.16 eV, leading to *U*_eq_ of −0.08 V, which is in close agreement with the experimental *U*_eq_ of −0.10 V^5^.

For CO_2_RR to CO, the reaction pathway is believed to proceed via CO_2_ hydrogenation as step 1: CO_2_(g) +H^+^ + e^−^ + * → *COOH, followed by *CO formation as step 2: *COOH + H^+^ + e^−^ → *CO + H_2_O(l), and *CO desorption as step 3: *CO → * + CO(g) (Fig. 4). Within the context of CHE^7,58^, the onset potential *U*_onset_ is given by the largest positive consecutive difference for step 1 and step 2 (i.e., −*U*_onset_ = max(Δ*G*_1_, Δ*G*_2_)/e^−^), as step 3 is not electrochemical. Here the solvation effects for surface species exposed to solvent water have been considered (see more details in Supplementary Methods 1.4 and Supplementary Table 6). As shown in Fig. 4, Δ*G*_1_ determines *U*_onset_ for all cases except for that on the Cu(100) surface predicted by PBE-D3BJ. On the Cu(111) surface, the predicted *U*_onset_ are −0.63 V, −0.36 V, and −0.75 V by XYG3:PBE-D3BJ, PBE-D3BJ and PBE, respectively, vs the relative hydrogen electrode (RHE). As the experimental value of *U*_onset_ is −0.60 V vs RHE^59^, the deviations from the experiment are -0.03 V (XYG3:PBE-D3BJ), 0.24 V (PBE-D3BJ) and −0.15 V (PBE), respectively. Similarly, on the Cu(100) surface, the deviations from the experimental *U*_onset_ of −0.30 V vs RHE^59^ are −0.09 V (XYG3:PBE-D3BJ), 0.24 V (PBE-D3BJ) and 0.04 V (PBE), respectively. Thus, the predicted *U*_onset_ of XYG3:PBE-D3BJ are in excellent agreement with the experiments. While the PBE results are reasonably good, the PBE-D3BJ results show large deviations.

It is quite alarming to see that the widely used PBE-D3BJ makes a wrong prediction on the potential limiting step, mainly due to the fact that it erroneously predicts a 0.26 eV more stable COOH in the gas phase (Supplementary Table 7). This error is diminished by chance in PBE for the lack of dispersion correction, leading to a reasonably good performance. However, both PBE and PBE-D3BJ provide a wrong relative stability of intermediates on Cu(100), while PBE-D3BJ yields a highly over-binded CO (Fig. 4), leading to an unrealistic tendency of catalyst poisoning. All in all, the hybrid XYG3:PBE-D3BJ scheme accurately describe both *U*_eq_ and *U*_onset_ for CO_2_RR to CO on both Cu(111) and Cu(100) surfaces, overcoming the shortcomings of PBE-D3BJ and PBE. These results highlight the importance of accurate description on the gas-phase energetics in understanding heterogeneous catalysis, in addition to the energetics of surface processes (Fig. 1). Nevertheless, the gas-phase correction schemes^7,22,60^, which represents one step towards the accurate description of the reaction energy profile for heterogeneous catalysis, are only useful when the description of the gas-phase reaction is in serious errors, while that of the surface reaction is already satisfactory (See additional examples and discussions in Supplementary Discussion and Supplementary Figs. 6 and 7). Thus, a method both accurate for the gas-phase and surface reactions, such as XYG3:GGA scheme, is highly desired to provide an unbiased understanding and prediction on heterogeneous catalysis.

Besides the applications to CO2RR to CO, the XYG3:PBE-D3BJ scheme is also applied to the preliminary exploration of the C–C coupling on the Cu(100) surface (Supplementary Fig. 8). Until now, the potential-determining step for the C_2+_ production is still under debate^61–63^. To validate the potential-determining step and to predict the selectivity for C_2+_ production, a comprehensive characterization of the whole reaction network is required. In addition, extending the application of XYG3:PBE-D3BJ to an accurate description of surface reactions on the other coinage metals is readily available. For instance, the XYG3:PBE-D3BJ scheme can accurately predict the HCOO* decomposition barrier on the Au(110)-1×2 surface, as opposed to the PBE-D3BJ method (Supplementary Fig. 7). However, for transition metals with opened d shells, such as Pt, it remains challenging to accurately describe the related chemisorption bonds and surface reactions, while more efforts have to be taken in developing new strategies with a suitable combination of H and L levels of theory on top of a suitable cluster model for the active center.

In summary, by using a hybrid scheme that combines the doubly hybrid XYG3 functional and the periodic GGA (i.e., XYG3:GGA), accurate first-principles calculations on copper-based heterogeneous catalysis have been achieved. The accuracy of the XYG3:PBE-D3BJ scheme was demonstrated by the benchmark study that includes the CO preferred sites, adsorption energies of CO, H, O, and NH_3_ and activation barriers for H_2_ dissociative adsorption and desorption on Cu(111) or Cu(100) surfaces. When the XYG3:PBE-D3BJ scheme was applied to the electrocatalytic CO_2_RR to CO on Cu(111) and Cu(100) surfaces, substantial improvements over the widely used PBE-D3BJ and PBE functionals on the calculated equilibrium and onset potentials have been achieved. The XO framework, powered by efficient algorithms of energies, gradient, and Hessians, provides a way to combine the best of different worlds. We anticipate that the easy use of the hybrid H:L scheme shown here, with H = XYG3 and L = PBE-D3BJ specifically for copper-based catalysts, as well as other suitable combinations of H and L in general, will boost the predictive power of periodic models for an accurate description of molecule-surface interactions in heterogeneous catalysis, and open up new horizons for the rational design of better industrial catalysts and new electrocatalytic materials.

## Methods

### DFT calculations

All periodic density functional theory (DFT) calculations were performed using the Vienna ab initio simulation package (VASP)^64,65^. The core electrons were described by the projector augmented-wave (PAW) method. Unless otherwise stated, the kinetic energy cutoff for the plane wave basis sets of the valence electrons was set to be 450 eV. The surface Monkhorst–Pack meshes^66^ of 5 × 5 × 1 k-point sampling in the surface Brillouin zone were employed for all calculations. After the convergence criteria for optimizations were met, the largest remaining force on each atom was less than 0.02 eV Å^‒1^. The climbing image nudged-elastic band (CI-NEB) method was employed to determine the transition states^67^.

All adsorption energy calculations using cluster models were performed by using the Q-Chem 5.0 computational package^68^. All the structures of cluster models cut from extended systems were fixed. The cluster model calculations were performed with a small basis set of def2-SVP^69^. For calculating the formation energy of the surface species in the gas phase, the large basis set of def2-QZVP^69^ was used. More details and discussions can be found in the Supplementary Methods.

### Thermodynamic quantity calculations

All gas-phase molecules were treated as ideal gas, whose thermodynamic quantities contain all the transitional, rotational, and vibrational contributions. All surface species were treated as an immobile model containing the vibrational contribution only. The thermodynamic quantities of the gas-phase molecules can be directly obtained from the Q-Chem calculation results with the vibrational contribution treated by the harmonic oscillator approximation. A brief introduction of partition functions also has been shown in the Supplementary Methods.

### Computational hydrogen electrode model

The Gibbs free energy change (*ΔG*) of the proton-coupled electron transfer (PCET) step was calculated by using the computational hydrogen electrode (CHE) model^7,58^, which uses one-half of the chemical potential of hydrogen as the chemical potential of the proton-electron pair. According to this method^7,58^, the Δ*G* value was determined as:

7 $$\varDelta G = \varDelta H-T\varDelta S+\varDelta {G}_{U}+\varDelta {G}_{{{{{{\rm{pH}}}}}}},$$

where Δ*H* and Δ*S* are the enthalpy change and the entropy change, respectively. *T* is the absolute temperature. Δ*G*_*U*_ is the free energy contribution related to the electrode potential *U*, where the effect of a bias on all states involving an electron in the electrode is included by shifting the energy of this state by −e*U*. Δ*G*_pH_ is the concentration correction to the H^+^ free energy. Since the values versus the relative hydrogen electrode (RHE) were used in this work, this correction was not necessary.

### Solvation model

The solvation effect was taken into account by using an implicit solvation model^70,71^ as implemented by the Hening group under the name VASPsol^72^, where a dielectric constant of 78.4 corresponds to solvent water. In addition, in the cases where there are strong interactions between the adsorbate and the water molecules, for instance, to form hydrogen bonds, it is necessary to add some explicit water molecules for a proper description of the solvation effect. Here, we suggested that explicit water molecules should be introduced, if the explicit solvation energy correction could provide additional stability for the adsorbate. More details and the suitability of our hybrid explicit–implicit solvation model can be found in the Supplementary Methods.

## Supplementary information

Supplementary Information

## Data Availability

The data that support the findings of this study are available from the corresponding author upon reasonable request.
