# Supplementary material for: Accurate descriptions of molecule-surface interactions in electrocatalytic CO2 reduction on the copper surfaces
Source: Nat Commun. 2023 Feb 20;14:936. doi: 10.1038/s41467-023-36695-7 (PMC9941474; doi:10.1038/s41467-023-36695-7)
Supplement: Supplementary file 1 — Supplementary Information [file 41467_2023_36695_MOESM1_ESM.pdf]

Supplementary Information

Accurate descriptions of molecule-surface interactions in electrocatalytic CO_2 reduction on the copper surfaces

Zheng Chen[1,3], Zhangyun Liu[1,3], Xin Xu[1,2]*

[1]Collaborative Innovation Center of Chemistry for Energy Materials, Shanghai Key Laboratory of Molecular Catalysis and Innovative Materials, MOE Key Laboratory of Computational Physical Sciences, Department of Chemistry, Fudan University, Shanghai 200433, People's Republic of China. [2]Hefei National Laboratory, Hefei 230088, P. R. China. [3] These authors contributed equally: Zheng Chen, Zhangyun Liu.

*E-mail: xxchem@fudan.edu.cn

Table of Contents

Supplementary Methods

1.1 DFT calculations

1.2 Thermodynamic quantity calculations

1.3 Computational hydrogen electrode model

1.4 Solvation model

Supplementary Discussion

Testing the accuracy by using the gas-phase correction schemes.

Supplementary Figures and Tables

Supplementary Fig. 1 Testing on errors of DFT methods and basis sets.

Supplementary Fig. 2 Testing on the cluster size effects

Supplementary Fig. 3 Testing on the cluster size effect with the hybrid (XYG3:PBE-D3BJ) scheme on the predicted CO adsorption energy on Au(111).

Supplementary Fig. 4 Testing on delocalization errors of selected DFT methods.

Supplementary Fig. 5 The efficiency of the cluster model calculations with PBE-D3BJ, B3LYP-D3BJ and XYG3 using the def2-SVP basis set.

Supplementary Fig. 6 Performance of the gas-phase (GP) correction schemes in predicting the formation energy of HCOO* on Cu(111).

Supplementary Fig. 7. Applying the XYG3:PBE-D3BJ scheme to the formate decomposition on the Au(110)-1×2 surface.

Supplementary Fig. 8. Free energy diagrams for the formation of *COCHO and *COCOH on the Cu(100) electrodes.

Supplementary Table 1 Experimental reaction enthalpies and electronic energies for selected gas phase reactions

Supplementary Table 2 Experimental electronic energies for selected surface reactions

Supplementary Table 3 Computed CO adsorption energies on different sites of Cu(111) surfaces.

Supplementary Table 4 CO frontier orbital energies

Supplementary Table 5 Computed adsorption energies for *CO, *H, *O on the Cu(111) surface and *NH3 on the Cu(100) surface.

Supplementary Table 6 Solvation energy corrections for *COOH and *CO on Cu(111) and Cu(100) surfaces.

Supplementary Table 7 Gas phase formation energy of COOH and HCOO.

Supplementary Reference

Supplementary Methods

1.1 DFT calculations. All periodic density functional theory (DFT) calculations were performed using Vienna *ab initio* simulation package (VASP).[1–3] The core electrons were described by the projector augmented-wave (PAW) method.[4] Unless otherwise stated, the kinetic energy cutoff for the plane wave basis sets of the valence electrons was set to be 450 eV. The surface Monkhorst–Pack meshes[5] of 5 × 5 × 1 k-point sampling in the surface Brillouin zone were employed for all calculations. A four-layered slab was adopted to model the 4 × 4 supercell of either Cu(111) or Cu(100) surfaces. All adsorbed species along with the top two layers of the slab were allowed to relax during energy minimization and minimum energy path calculations, while the bottom two layers of the slab were kept fixed at the bulk positions. In order to avoid spurious interactions between atoms in neighboring slabs, a vacuum layer of 20 Å was added in the direction perpendicular to the surface. After the convergence criteria for optimizations were met, the largest remaining force on each atom was less than 0.02 eV $Å^{-1}$. The climbing image nudged-elastic band (CI-NEB) method was employed to determine the transition states.[6,7] The generalized gradient approximation (GGA) of the Perdew–Burke–Ernzerhof (PBE) functional[8] was used. The contributions of dispersive interactions were accounted for by using the DFT+D3 method with Becke-Jonson (BJ) damping.[9,10]

All adsorption energy calculations using cluster models were performed by using the Q-Chem 5.0 computational package.[11] All the structures of cluster models cut from extended systems were fixed. The cluster model calculations were performed with a small basis set of def2-SVP.[12] Here we refer to **Supplementary Fig. 2** for an illustrative testing on the cluster size effects for CO adsorption on Cu(111) and Cu(100) surfaces. In addition, by using the calculations of *CO on the Cu_{31} cluster as an illustrative example, a direct efficiency comparison among PBE-D3BJ, B3LYP-D3BJ and XYG3 is provided in **Supplementary Fig. 5**. Although the detailed results may vary from systems to systems, the results show that the time cost of XYG3 is at the same order of PBE-D3BJ and B3LYP-D3BJ. All of them are very

efficient, as the current hybrid scheme allows the using of a small basis set to capture the adsorption energy difference from the H and L levels (Eq. 5 in the main text).

For calculating the formation energy of the surface species in the gas phase, the reactant molecule used as the reference was optimized by the corresponding high level method, except for the CCSD(T) calculation, where the structure was optimized by the XYG3[13,14] functional. The structure of the surface species was frozen as it was on the surface. The large basis set of def2-QZVP[12] was used. For calculating the reaction energy in **Supplementary Fig. 1**, all molecular structures were fully relaxed using the corresponding methods and the specified basis sets.

1.2 Thermodynamic quantity calculations. All gas phase molecules were treated as an ideal gas, whose thermodynamic quantities contain all the transitional, rotational and vibrational contributions. All surface species were treated as an immobile model containing the vibrational contribution only.[15] In the following, we briefly introduce the partition functions for the transitional, rotational and vibrational contributions, as well as how the internal thermal energy *U*, enthalpy *H* and Gibbs free energy *G* were calculated using the partition functions. Also, the thermodynamic quantities of the gas phase molecules can be directly obtained from the Q-Chem calculation results with the vibrational contribution treated by the harmonic oscillator approximation.

For partition functions of species A, all the transitional, rotational and vibrational contributions from the gas phase were considered. Treating A as an ideal gas, its transitional and rotational partition functions Q_A^{trans} and Q_A^{rot} were calculated by[15]

$$Q_A^{trans} = \left(\frac{2\pi m_A k_B T}{h^2}\right)^{\frac{3}{2}} V \quad \text{(S1)}$$

$$Q_A^{rot} = \frac{1}{\sigma^*}\frac{k_B T}{hB^{rot}} \text{(Linear)}; \qquad Q_A^{rot} = \frac{1}{\sigma^*}\left(\frac{k_B T}{h}\right)^{3/2}\sqrt{\frac{\pi}{A^{rot}B^{rot}C^{rot}}} \text{ (nonlinear)} \quad \text{(S2)}$$

Here $V = \frac{k_B T}{p_A}$ is the volume of the system, σ^* is the symmetry factor, A^{rot}, B^{rot}, C^{rot} are the rotational constants, and *h* is the Plank's constant. Harmonic oscillator

approximation was applied to both A and A*, where the vibrational partition function Q^{vib} was calculated according to [15]

$$Q^{vib} = \prod_i \frac{e^{-\frac{hv_i}{2k_BT}}}{1-e^{-\frac{hv_i}{k_BT}}} \tag{S3}$$

Here v_i refers to the frequency of the *i*th mode.

The internal thermal energy *U* was obtained from the total partition function *Q* by

$$U = k_B T^2 \left(\frac{\partial \ln Q}{\partial T}\right)_V \tag{S4}$$

After *U* was obtained, the enthalpy *H* was calculated as

$$H = U + PV \tag{S5}$$

Usually, the *PV* term was considered for the gas phase species only. For an ideal gas, *PV* was taken as *RT* per mol gas. Finally, the Gibbs free energy *G* was given by

$$G = -RT \ln Q + PV \tag{S6}$$

1.3 Computational hydrogen electrode model. The Gibbs free energy change (ΔG) of the proton-coupled electron transfer (PCET) step was calculated by using the computational hydrogen electrode (CHE) model,[16,17] which uses one-half of the chemical potential of hydrogen as the chemical potential of the proton-electron pair. According to this method, [16,17] the ΔG value was determined as:

$$\Delta G = \Delta H - T\Delta S + \Delta G_U + \Delta G_{pH}, \tag{S7}$$

where ΔH and ΔS are the enthalpy change and the entropy change, respectively. *T* is the absolute temperature. ΔG_U is the free energy contribution related to the electrode potential *U*, where the effect of a bias on all states involving an electron in the electrode is included by shifting the energy of this state by $-eU$. ΔG_{pH} is the concentration correction to the H^+ free energy. Since the values versus the relative hydrogen electrode (RHE) were used in the present work, this correction was not

necessary. During calculations, for convenience, we assumed the chemical potential of water in solution was equal to 3.169 kPa, the same as pure liquid water at room temperature.

The thermodynamic free energy diagrams were then established by using Eq. S7. In this approach, the onset potential U_{onset} is numerically equivalent to the additive inverse of the largest positive reaction energy of all PCET steps on the free energy diagram.

1.4 Solvation model. Solvation effect was taken into account by using an implicit solvation model[18,19] as implemented by the Hening group under the name VASPsol,[20] where a dielectric constant of 78.4 corresponds to solvent water. Using adsorbate A* as an example, the formation free energy of A* in the gas phase ($\Delta G_{A*(g)}$) and that in the solution phase ($\Delta G^{\text{im}}_{A*(aq)}$) are respectively given by

$$\Delta G_{A*(g)} = G_{A*(g)} - G_{*(g)} - G_{A(g)}, \tag{S8}$$

$$\Delta G^{\text{im}}_{A*(aq)} = G^{\text{im}}_{A*(aq)} - G^{\text{im}}_{*(aq)} - G_{A(g)}, \tag{S9}$$

where $G_{A*(g)}$, $G_{*(g)}$ are the total energies for adsorbate A* and the surface site in the gas phase, while $G^{\text{im}}_{A*(aq)}$, $G^{\text{im}}_{*(aq)}$ are the corresponding total energies in the solution phase. Therefore, the formation energy of A* in the solution phase described with an implicit solvent model is given by

$$\Delta G^{\text{im}}_{A*(aq)} = \Delta G_{A*(g)} + (G^{\text{im}}_{A*(aq)} - G_{A*(g)}) - (G^{\text{im}}_{*(aq)} - G_{*(g)}). \tag{S10}$$

The last two terms in the right hand side of equation S10 are defined as the implicit solvation energy correction term $\Delta G^{\text{im-corr}}$. Assuming that the vibration contributions are similar in the gas phase and the solution phase, the $\Delta G^{\text{im-corr}}$ term is simplified to the corresponding electronic energy term $\Delta E^{\text{im-corr}}$.

In the cases where there are strong interactions between the adsorbate and the water molecules, for instance, to form hydrogen bonds, it is necessary to add some explicit water molecules for a proper description of the solvation effect. Here, we suggested that explicit water molecules should be introduced, if the explicit solvation

energy correction could provide an additional stability for the adsorbate (i.e., $\Delta G^{\text{ex-corr}} < 0$),

$$\Delta G_{A^*(aq)}^{ex-corr} = G_{A^*\bullet nH_2O(aq)}^{\text{im}} - G_{*(aq)}^{\text{im}} - nG_{H_2O(aq)} \,. \tag{S11}$$

In the present work, the total solvation energy corrections include both $\Delta G^{\text{im-corr}}$ and $\Delta G^{\text{ex-corr}}$, while the latter for describing the hydrogen bonds is updated to the CCSD(T) level from the PBE-D3BJ level in the gas phase.

We briefly discussed the suitability of our hybrid explicit-implicit solvation model. As is well known, ab initio molecular dynamics (AIMD) simulations contain explicit solvents, which, in principle, can provide a more accurate description for solvation effect, albeit at a higher computational cost. To gain efficiency, an alternative way is to use the implicit solvation models, where the solvent is represented as a homogeneous dielectric constant surrounding the solute. In this way, the continuum solvation models consider thermally averaged solvent molecules, which lead to a model with only a small number of parameters used to represent the solvent with reasonable accuracy in many situations.[21]

A key limitation of the implicit solvation models is the unsatisfactory description of the site-specific interactions between the solute and the solvents (e.g., hydrogen bonding). To address the challenge of the site-specific interactions in continuum solvation models, mixed continuum models are often adopted, in which in addition to the implicit solvent, a few (usually one or two) solvent molecules are explicitly included to the ab initio description of the reaction system to better characterize the site-specific interactions such as hydrogen bonding.[22] This approach, dubbed as *microsolvation*,[22,23] has been successfully applied for the accurate prediction of the pKa values of mono and polyprotic acids in the aqueous solution and was concluded to be accurate whenever the explicit solvent–solute interaction is strong enough, such that it can be well described within the harmonic approximation.[24,25]

Very recently, Heenen *et al.*[26] performed a benchmark study for the solvation effects at the uncharged metal/water interfaces via intensive AIMD simulations. As shown in **Supplementary Table 6**, the solvation energies predicted by our hybrid

explicit-implicit solvation model agree well with the AIMD results. Therefore, according to both the understanding of the physical picture and the performance results, we believe that the hybrid explicit-implicit solvation model used in this work can provide reasonable solvation energies for CO_2RR on Cu(111) and Cu(100) surfaces.

Supplementary Discussion

Testing the accuracy by using the gas-phase correction schemes. It is well-known that GGAs perform poorly in the prediction of reaction energies and reaction barriers in the gas phase.[13,27–29] Thus, the gas-phase correction schemes have been developed to remove the gas-phase errors,[17,29,30] which represents one step towards the accurate description of the reaction energy profile for heterogeneous catalysis. Here we use the formation energy of HCOO* on Cu(111) as an illustrative example. HCOO* is quite possibly involved as an important intermediate in many reactions such as the methanol synthesis and the water-gas shift reaction.[31,32] A combination of the experimental adsorption energy of HCOO*[33] on the Cu(111) surface and the CCSD(T) calculated formation energy of HCOO(g) for both proper descriptions of the gas phase and the surface reactions will be used as a plausible reference for benchmarking.

Two types of the gas-phase correction schemes are used. The first one is directly adding a correction term to the energy of a specific gas-phase molecular species.[17,29,30] With these gas-phase corrections, the large errors for reaction energies in gas-phase predicted by GGAs can be significantly reduced.[17,29] For the PBE functional, the gas-phase corrections of CO_2 and H_2 were determined as 0.13 eV and -0.08 eV respectively, by a gas-phase data set with 11 species in 21 reactions.[17,30] Similarly, another set of gas-phase corrections for CO_2 and H_2 were determined as 0.19 eV and 0.00 eV, respectively, using a different data set including 27 products from CO_2 and H_2.[29] These gas-phase (GP) correction methods are considered as semi-empirical,[29] as they are fitted to a set of species in some chosen reactions, which are denoted as "GP correction-1" and "GP correction-2", respectively, here. Certainly, CCSD(T) provides

a none-empirical way to correctly describe the gas-phase reactions, which is denoted as “GP correction-3”. Since the gas-phase reaction energies from small molecules predicted by PBE and PBE-D3BJ are almost the same, the above two sets of GP corrections developed for PBE are employed to correct the PBE-D3BJ results here. We then use the corrected formation energy of gas-phase HCOO from CO_2 and H_2, which is then combined with the PBE-D3BJ adsorption energy to give the formation energy of surface HCOO* for PBE-D3BJ.

As shown in **Supplementary Fig. 6**, XYG3:PBE-D3BJ leads to a good result, which compares well with the benchmark value, as both gas-phase and surface reactions have been properly described by XYG3:PBE-D3BJ. As shown in **Supplementary Fig. 6**, PBE-D3BJ also leads to a reasonably good result, despite its large error in predicting the adsorption energy of HCOO* (**Fig. 2c** in the manuscript). This is because that the large errors in HCOO* adsorption energy (**Fig. 2c**) and the gas-phase HCOO formation energy (**Supplementary Table 7**) are cancelled out by chance for PBE-D3BJ. Thus, introducing only the gas-phase corrections could diminish this error cancellation to show even larger deviations from the reference. Here for the HCOO* formation energy, when the error for the gas-phase reaction is perfectly corrected by CCSD(T), the results of “GP correction-3” show an error as large as 0.50-0.68 eV. Therefore, the gas-phase correction schemes are only useful to improve the accuracy, when the description of the gas-phase reaction is in serious errors, while that of the surface reaction is already satisfactory. To guarantee the accuracy and to go beyond error cancellation, both the gas phase and the surface reaction should be properly described as shown by the XYG3:PBE-D3BJ scheme.

In addition, the previous gas-phase correction schemes[17,29,30] will yield the same correction values for the initial state and the transition state for an elementary surface reaction. Thus the energy barrier, usually poorly predicted by the GGA functionals, cannot be improved by using the previous gas-phase correction schemes. For instance, an experimentally established activation energy for the formate decomposition on the Au(110)-1x2 surface has been reported very recently,[34] which can serve as a

benchmark to validate the theoretical method on calculating the barrier of an elementary reaction. As shown in **Supplementary Fig. 7**, our hybrid scheme predicts the activation energy of 1.08 eV, which is in good agreement with the experimental result of 1.03±0.02 eV, while the corresponding value from PBE-B3DJ is 0.78 eV. Therefore, our hybrid scheme can promote the accuracy on describing the related catalytic systems by providing more accurate DFT calculation results on the elementary reactions.

Supplementary Figures and Tables

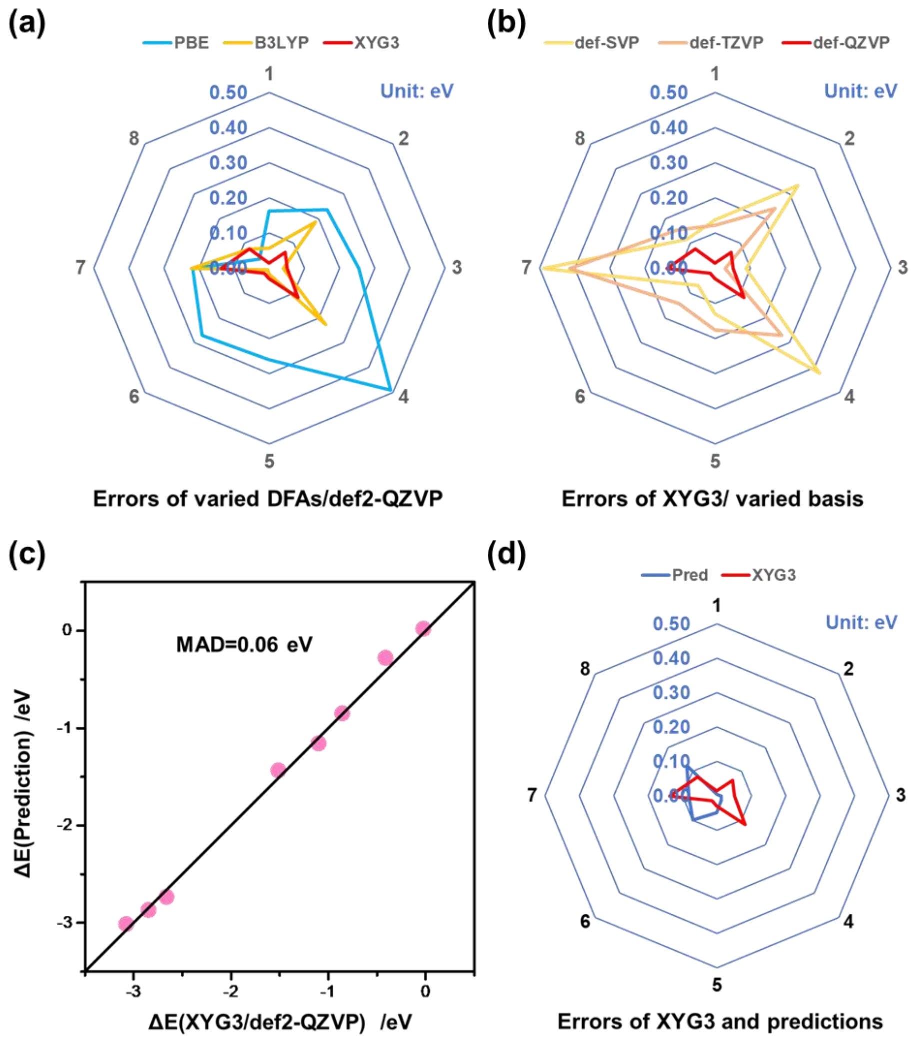

Supplementary Fig. 1 | Testing on errors of DFT methods and basis sets. Eight gas phase reaction energies are listed in **Supplementary Table 1**, which are related to some net reactions commonly pursued in heterogeneous catalysis. The corresponding experimental values are used as the references. (**a**) The absolute errors of PBE, B3LYP and XYG3 functionals, respectively, with large basis set of def2-QZVP. (**b**) The absolute errors of the XYG3 functional with basis sets of def2-SVP, def2-TZVP and def2-QZVP, respectively. (**c**) Comparison on the reaction energies predicted by a combined method using {PBE/def2-QZVP + (XYG3/def2-SVP – PBE/def2-SVP)} to approach the targeted values from XYG3/def2-QZVP. (**d**) The absolute errors between the predicted (Pred) number and the corresponding targeted number of XYG3/def2-QZVP. It is demonstrated here that the prediction with the combined method do not reduce the accuracy of XYG3 for reproducing the experimental values.

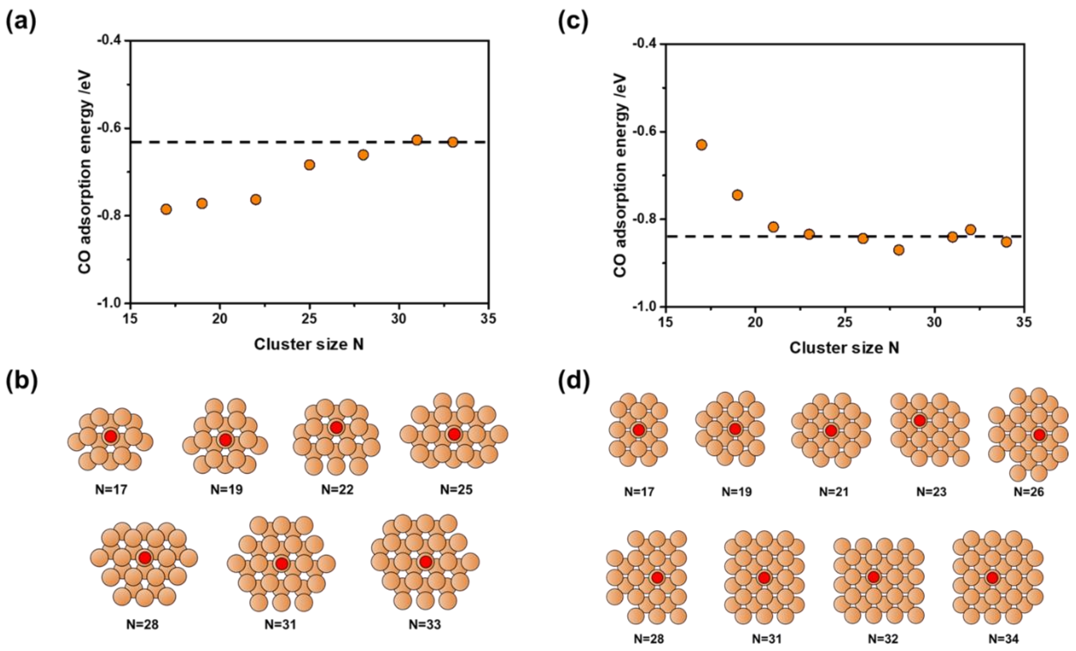

Supplementary Fig. 2 | Testing on the cluster size effects with the hybrid (XYG3:PBE-D3BJ) scheme. **(a)** The influences of cluster size on the predicted CO adsorption energies on Cu(111), and **(b)** the corresponding structure of the clusters. **(c)** The influences of cluster size on the predicted CO adsorption energies on Cu(100), and **(d)** the corresponding structure of the clusters. In **(b)** and **(d)**, the orange balls represent Cu atoms, while the red balls represent *CO. Appropriate shapes of clusters are chosen with the lowest spin states to be compatible with the bulk copper.

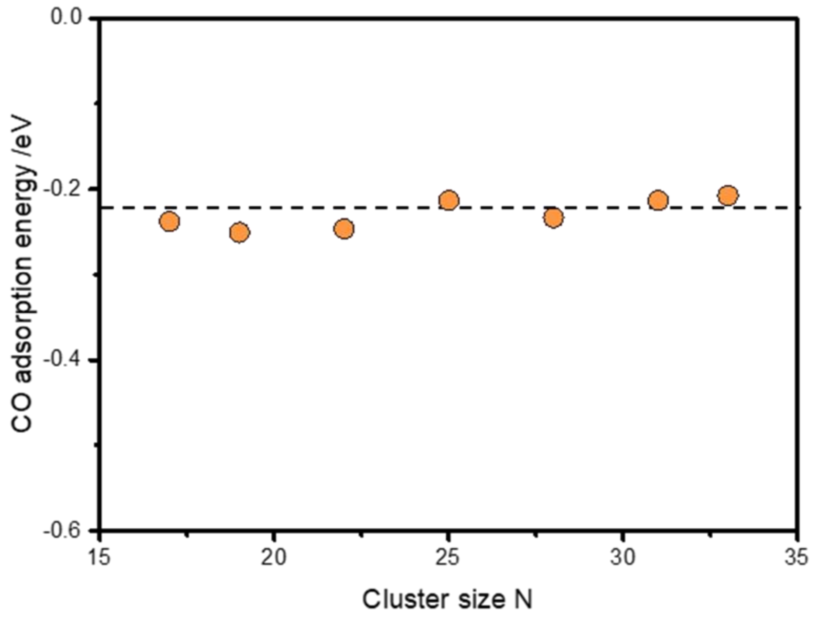

Supplementary Fig. 3 | Testing on the cluster size effect with the hybrid (XYG3:PBE-D3BJ) scheme on the predicted CO adsorption energy on Au(111). The corresponding structure of the clusters are the same as those of Cu(111) shown in **Supplementary Fig. 2**. Appropriate shapes of clusters are chosen with the lowest spin states to be compatible with the bulk gold.

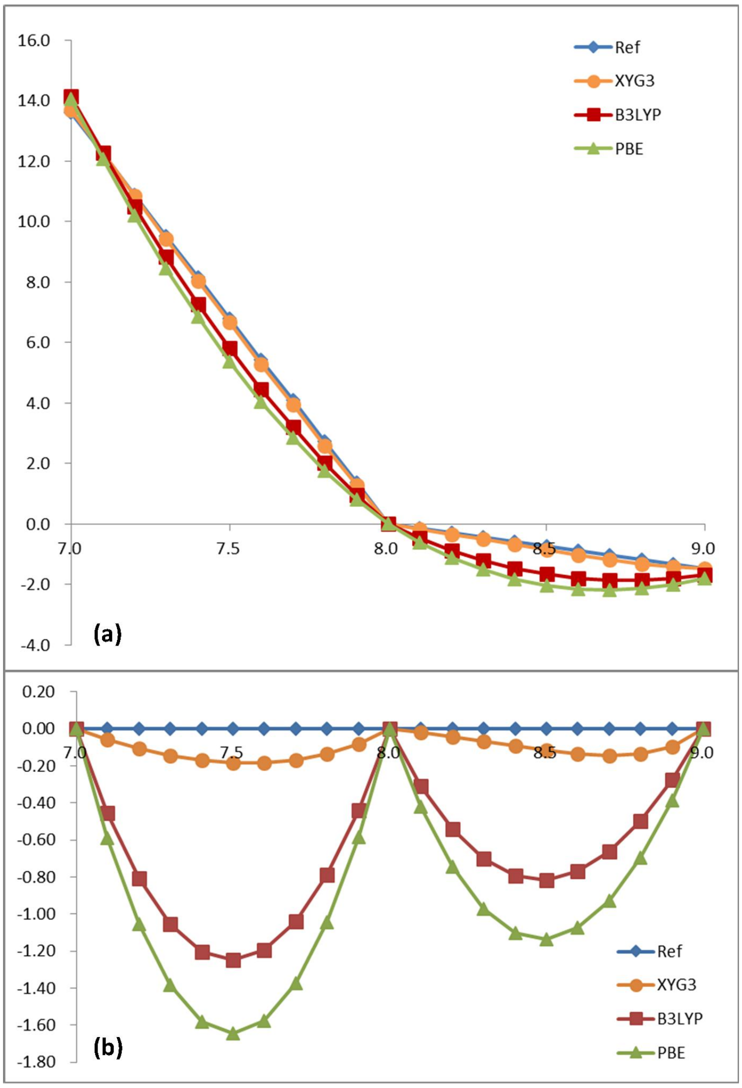

Supplementary Fig. 4 | Testing on delocalization errors of selected DFT methods. (a) Fractional charge behaviors for the oxygen atom with methods of PBE, B3LYP, and XYG3. Note that the dispersion correction will make no contribution. The exact straight lines, denoted as Ref, are obtained using the experimental IP and EA of the oxygen atom. (b) Deviations from the corresponding linear interpolations for the oxygen atom with methods of PBE, B3LYP, and XYG3.

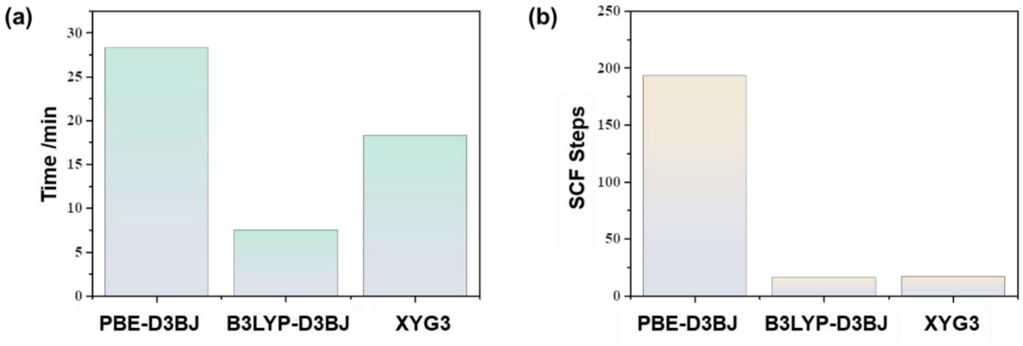

Supplementary Fig. 5 | The efficiency of the cluster model calculations with PBE-D3BJ, B3LYP-D3BJ and XYG3 using the def2-SVP basis set. (a) The time cost of the respective calculation for *CO on the Cu_{31} cluster cut from the Cu(111) surface. (b) The corresponding steps used for reaching the self-consistent field (SCF) convergence. All calculations are carried out on a 3.0-GHz Intel Xeon 6154 machine.

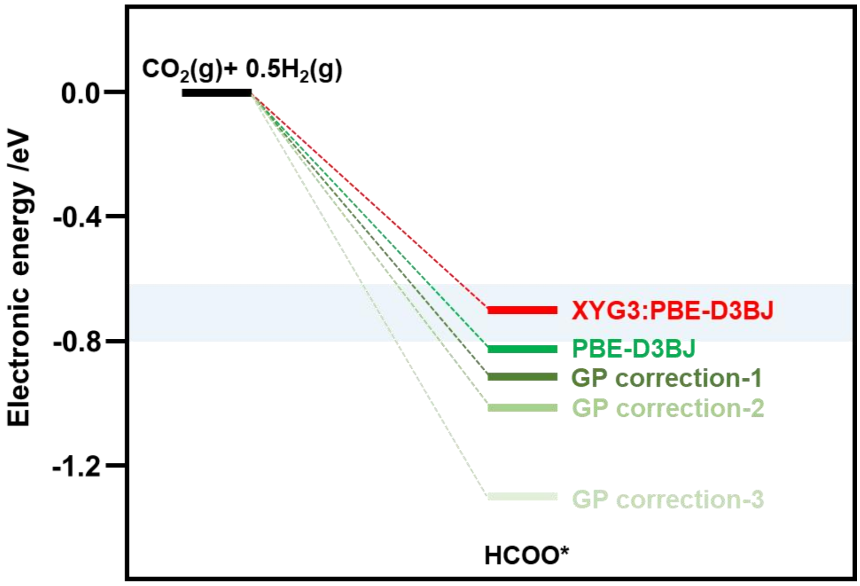

Supplementary Fig. 6 | Performance of the gas-phase (GP) correction schemes in predicting the formation energy of HCOO* on Cu(111). The horizontal bar in light blue represents the reference, which combines the experimental adsorption energy of HCOO* and the CCSD(T) formation energy of HCOO(g). For "GP correction-1"[17] and "GP correction-2",[29] the semiempirical gas-phase correction schemes proposed in previous studied are employed to correct the PBE-D3BJ results. "GP correction-3" is non-empirical, where the gas-phase errors are corrected by using CCSD(T).

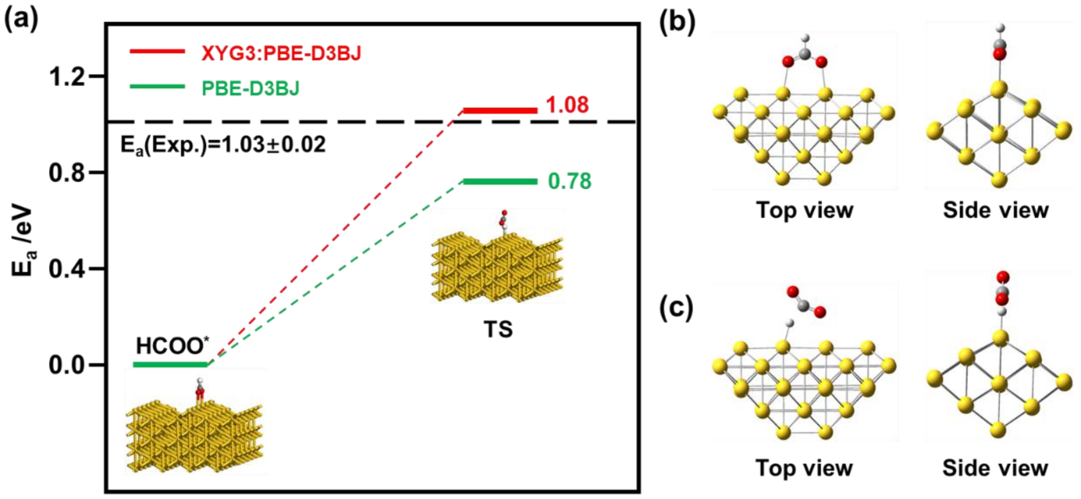

Supplementary Fig. 7 | Applying the XYG3:PBE-D3BJ scheme to the formate decomposition on the Au(110)-1×2 surface. (a) Activation barrier (Ea) calculated by XYG3:PBE-D3BJ (red) or PBE-D3BJ (green). The dash line is the experimental result, where the activation energy was determined by the heating rate variation analysis at the formate coverage of 0.04 ML.[34] The top view and the side view of the cluster model for the active center, employed in the XYG3:PBE-D3BJ hybrid scheme for (b) the HCOO* adsorption and (c) the decomposition transition state (TS).

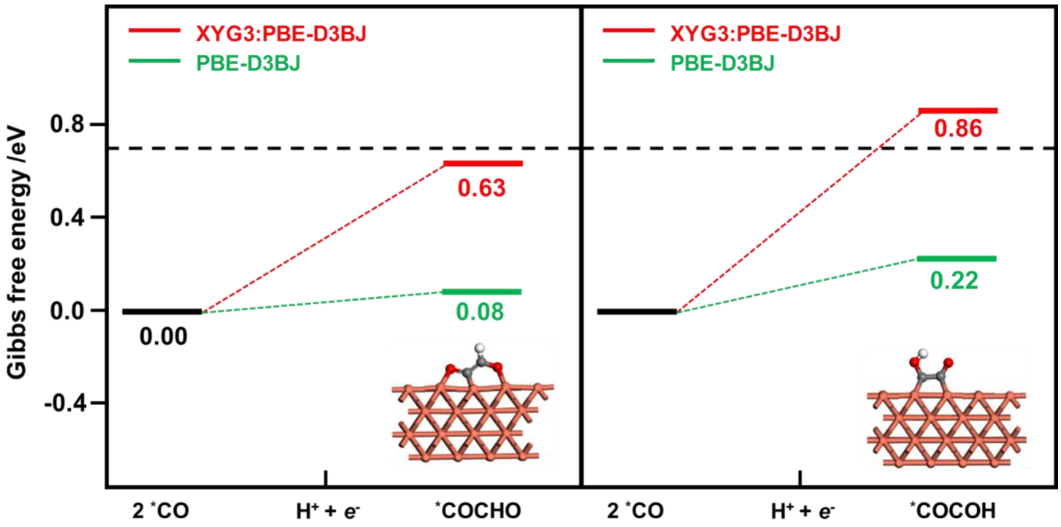

Supplementary Fig. 8 | Free energy diagrams for the formation of *COCHO and *COCOH on the Cu(100) electrodes. The red line and the green line correspond to the values predicted by XYG3:PBE-D3BJ and PBE-D3BJ, respectively. The black dashed line at 0.70 eV marks the free energies corresponding to the experimental onset potential U_{onset} of C_2H_4 on the Cu(100) surface (−0.70 V vs the relative hydrogen electrode (RHE)).[35] Although the formation free energies of *COCHO and *COCOH predicted by XYG3:PBE-D3BJ are close to the experimental onset potential U_{onset} of C_2H_4 on the Cu(100), we would like to point out that the corresponding potential-determining step is still under debate.[36–38]

Supplementary Table 1 Experimental reaction enthalpies **ΔH** and electronic energy changes **ΔE** for selected gas phase reactions, in relation to some net reactions commonly pursued in heterogeneous catalysis. Unit: eV

Number	Reactions	**ΔH** [a]	**ΔH^corr** [b]	**ΔE** [c]
**1**	$1.5H_2(g)+0.5N_2(g) \rightarrow NH_3(g)$	-0.48	0.37	-0.84
**2**	$H_2(g)+0.5O_2(g) \rightarrow H_2O(g)$	-2.51	0.22	-2.73
**3**	$CO(g)+0.5O_2(g) \rightarrow CO_2(g)$	-2.93	0.09	-3.02
**4**	$CO(g)+H_2O(g) \rightarrow CO_2(g)+H_2(g)$	-0.43	-0.13	-0.30
**5**	$CO(g)+3H_2(g) \rightarrow CH_4(g)+H_2O(g)$	-2.14	0.68	-2.82
**6**	$CO(g)+2H_2(g) \rightarrow CH_3OH(g)$	-0.98	0.55	-1.53
**7**	$CO_2(g)+3H_2(g) \rightarrow CH_3OH(g)+H_2O(g)$	-0.55	0.68	-1.24
**8**	$CO_2(g)+H_2(g) \rightarrow HCOOH(g)$	0.15	0.26	-0.10

[a] Reaction enthalpy calculated by using the formation enthalpies of gas phase species at standard conditions, taken from the NIST web site.[39]

[b] Thermal correction to the enthalpy, which includes contributions from all translation, rotation, vibration and volumetric work, while the gas phase species is treated as an ideal gas using the harmonic oscillator approximation.

[c] Experimental electronic energy change obtained by **ΔH** - $\mathbf{\Delta H^{corr}}$.

Supplementary Table 2 Experimental electronic energies for selected surface reactions **ΔE_{exp}**, obtained from experimental enthalpies **ΔH_{exp}**, internal energies **ΔU_{exp}** and the calculated zero-point energy change **ΔZPE**.[a,b] Unit: eV

Number	Surface reactions	ΔH_{exp}	ΔU_{exp}	ΔZPE	ΔE_{exp}
**1**	CO(g)+Cu(111) → CO/Cu(111)	-0.55	-0.54	0.05	-0.59
**2**	NH_3(g)+Cu(100) → NH_3/Cu(100)	-0.59	-0.57	0.05	-0.62
**3**	0.5O_2(g)+Cu(111) → O/Cu(111)	-2.02	-2.00	0.08	-2.08
**4**	2H/Cu(111) → TS(H-H)/Cu(111)	0.77	0.77	-0.07	0.84
**5**	HCOO(g) +Cu(111) → $HCOO_{bi}$/Cu(111)	-3.49~ -3.31	-3.47~ -3.29	0.13	-3.60~ -3.42
**6**	H_2(g)+Cu(111) → TS(H-H)/Cu(111)	/	/	/	0.63
**7**	0.5H_2(g)+Cu(111) → H/Cu(111)	/	/	/	-0.10

[a] Reaction enthalpy **ΔH_{exp}**, obtained directly from experiments, were converted to changes in internal energy **ΔU_{exp}** by adding a correction of -Δ*n*RT, where Δ*n* is the change of gas moles, RT is the Rydberg constant multiply temperature. The zero-point energy (**ZPE**) was calculated using the PBE functional with the harmonic approximation to get the normal-mode frequencies, which were used to estimate the **ZPE** change in a specific reaction (**ΔZPE**). Finally, **ΔE_{exp}** is defined as (**ΔU_{exp}** – **ΔZPE**), which can be directly compared to the calculated electronic energy change in the corresponding reaction.

[b] All **ΔH_{exp}**, **ΔU_{exp}** , **ΔZPE** and **ΔE_{exp}** of surface reactions **1** and **2** were taken from ref[40], where the original experiments were performed as in ref[41,42]. **ΔH_{exp}** of surface reaction **3** was from ref[43], while **ΔZPE** was calculated in the present work. **ΔH_{exp}** of surface reaction **4** was from ref[44], while **ΔZPE** was calculated in the present work. **ΔH_{exp}** of surface reaction **5** was from ref[33], while **ΔZPE** was calculated in the present work. For surface reaction **6**, the H_2 dissociation barrier of 0.63 eV calculated by a specific reaction parameter (SPR)-DFT was used here as an indirect experimental value,[45] as the SPR-DFT potential energy surface could well reproduce experimental data on the dissociative adsorption probability as a function of incidence energy and reactant state, as well as the data on rotationally inelastic scattering, with chemical accuracy. **ΔE_{exp}** of surface reaction **7** was obtained from the **ΔE_{exp}** of surface reactions **4** and **6**.

[c] For surface reactions **1**, the adsorption energy of CO was measured (0.52±0.02 eV) by isosteric heat of adsorption at coverage < 0.25 ML.[41] For surface reaction **2**, the desorption energy of NH_3 was measured (0.59±0.02 eV) by isothermal desorption at coverage < 0.15 ML.[42] For surface reaction **3**, the adsorption energy of O_2 was measured by microcalorimetry at the beginning of the O_2 adsorption, where a constant heat of adsorption (2.02 eV) was measured. [43] For surface reaction **4**, the desorption barrier of H* was measured by temperature programmed desorption.[44] The pre-factor ν and desorption activation energy E_{des} was determined by using the "complete

analysis" method[46] which allows the determination of the coverage dependent desorption energy using a complete set of desorption spectra. Over the coverage range from 0~0.2 ML, the desorption energy and pre-factor were determined as E_{des} = 0.77±0.01 eV and ln ν = 25.9±0.4 s^{-1}.[44] For surface reaction **5**, the adsorption energy of $HCOO_{bi}$ was measured (-3.49 ~ -3.31 eV) by adsorption calorimetry at coverage of 0~0.11 ML.[33] To match the low surface coverage where experimental values were measured, all the calculations were performed at low coverage of 0.06 ML.

Supplementary Table 3 Computed CO adsorption energies on different sites of Cu(111) surface.[a] Unit: eV

	**PBE**	**PBE-D3BJ**	**M06-L**	**B3LYP**	**B3LYP-D3BJ**	**XYG3**	**Exp.**
top	-0.75	-0.99	-0.71 (-0.65)[b]	-0.27	-0.69	-0.63	-0.59
bri	-0.79	-1.05	-0.74	-0.02	-0.49	-0.57	/
fcc	-0.85	-1.09	-0.75 (-0.70)[b]	0.16	-0.26	-0.51	/
hcp	-0.84	-1.08	-0.73	0.05	-0.38	-0.49	/

[a] The results of PBE and PBE-D3BJ were calculated directly with the periodic boundary condition using VASP. The results of other functionals (H) were calculated by using the hybrid (H:L) scheme introduced in the present work, where the low level method (L) was the periodic PBE-D3BJ. Top, bri, fcc and hcp stand for the top site, bridge site and hollow site (fcc, hcp), respectively.

[b] The values in the brackets were calculated by the periodic M06-L as reported in ref[47]

Supplementary Table 4 CO frontier orbital energies for the highest occupied molecular orbital (HOMO) and the lowest unoccupied molecular orbital (LUMO). Unit: eV

CO	Expt.[a]	RPA[b]	XYG3[c]	B3LYP	M06-L	PBE
$-\varepsilon_{HOMO}$	0.61	0.57	0.58	0.39	0.34	0.33
$-\varepsilon_{LUMO}$	-0.08	-0.08	-0.07	0.04	0.05	0.07
Δ_{KS}	0.69	0.65	0.65	0.35	0.29	0.26

[a] Experimental data taken from Refs. [48] and [49].

[b] RPA/G0W0@PBE data taken from Ref. [50].

[c] XYG3 orbital energies were calculated by the energy derivative with respect to the orbital occupation.

Supplementary Table 5 Computed adsorption energies for *CO, *H, *O on the Cu(111) surface and $*NH_3$ on the Cu(100) surface.[a] Unit: eV

	**PBE**	**PBE-D3BJ**	**M06-L**	**B3LYP**	**B3LYP-D3BJ**	**XYG3**	**Exp.**
*CO	-0.75	-0.99	-0.71	-0.27	-0.69	-0.63	-0.59
*H	-0.17	-0.26	-0.21	0.04	-0.11	-0.10	-0.10
*O	-1.54	-1.66	-1.52	-1.26	-1.45	-2.01	-2.08
$*NH_3$	-0.64	-0.87	-0.59	-0.40	-0.79	-0.74	-0.62
MAD[b]	0.20	0.31	0.21	0.38	0.23	0.06	/

[a] The results of PBE and PBE-D3BJ were calculated directly with the periodic boundary condition using VASP. The results of other functionals (H) were calculated by using the hybrid (H:L) scheme introduced in the present work, where the low level method (L) was the periodic PBE-D3BJ.

[b] Mean absolute deviation (MAD) was calculated using the absolute deviation from the corresponding experimental value.

Supplementary Table 6 Solvation energy corrections of adsorbates on Cu(111) and Cu(100) surfaces.[a] Unit: eV

	$\Delta G^{\text{im-corr}}$	$\Delta G^{\text{ex-corr}}$	$\Delta G^{\text{tot-corr}}$	ΔG^{AIMD}
Cu(111)-*COOH	-0.21	-0.17 [b]	-0.38	/
Cu(111)-*CO	-0.02	/ [c]	-0.02	0.01±0.06 [d]
Cu(111)-*OH	-0.13	-0.26 [b]	-0.39	-0.32±0.05 [d]
Cu(100)-*COOH	-0.21	-0.26 [b]	-0.47	/
Cu(100)-*CO	-0.02	/ [c]	-0.02	/
Cu(100)-*COCHO	-0.11	-0.28 [b]	-0.39	/
Cu(100)-*COCOH	-0.22	-0.30 [b]	-0.52	/

[a] More details for estimating the solvation energy corrections can be found in section **S1.4**.

[b] Two explicit water molecules are found to bind to *COOH by forming two hydrogen bonds with the -OH group (one as a donor and one as an acceptor), as suggested in a previous work [51]. This is also true for the -OH group of *COCOH. Three explicit water molecules are found to bind to *OH (two as donors and one as an acceptor). For *COCHO, only one explicit water molecule is found to bind to the -CHO group, which acts as a donor.

[c] The solvent water molecule is unable to bind to *CO to form a stable complex during the geometry optimization using the implicit solvation model.

[d] The ab initio molecular dynamics (AIMD) results were obtained from ref[26]

Supplementary Table 7 Formation energy of COOH and HCOO in the gas phase.[a] Unit: eV

	ΔE_f(COOH)	ΔE_f(HCOO)
PBE/PAW	2.31	2.19
PBE-D3BJ/PAW	2.30	2.19
CCSD(T)/def2-QZVP	2.56	2.80

[a] The frozen structure of COOH on the Cu(100) surface and that of HCOO on the Cu(111) surface are used respectively.

Supplementary References

1. Kresse, G. & Hafner, J. *Ab initio* molecular dynamics for open-shell transition metals. *Phys. Rev. B* **48**, 13115–13118 (1993).
2. Kresse, G. & Furthmüller, J. Efficiency of ab-initio total energy calculations for metals and semiconductors using a plane-wave basis set. *Comput. Mater. Sci.* **6**, 15–50 (1996).
3. Kresse, G. & Furthmüller, J. Efficient iterative schemes for *ab initio* total-energy calculations using a plane-wave basis set. *Phys. Rev. B* **54**, 11169–11186 (1996).
4. Blöchl, P. E. Projector augmented-wave method. *Phys. Rev. B* **50**, 17953–17979 (1994).
5. Monkhorst, H. J. & Pack, J. D. Special points for Brillouin-zone integrations. *Phys. Rev. B* **13**, 5188–5192 (1976).
6. Henkelman, G. & Jónsson, H. Improved tangent estimate in the nudged elastic band method for finding minimum energy paths and saddle points. *J. Chem. Phys.* **113**, 9978–9985 (2000).
7. Henkelman, G., Uberuaga, B. P. & Jónsson, H. A climbing image nudged elastic band method for finding saddle points and minimum energy paths. *J. Chem. Phys.* **113**, 9901–9904 (2000).
8. Perdew, J. P., Burke, K. & Ernzerhof, M. Generalized gradient approximation made simple. *Phys. Rev. Lett.* **77**, 3865–3868 (1996).
9. Grimme, S., Antony, J., Ehrlich, S. & Krieg, H. A consistent and accurate *ab initio* parametrization of density functional dispersion correction (DFT-D) for the 94 elements H-Pu. *J. Chem. Phys.* **132**, 154104 (2010).
10. Grimme, S., Ehrlich, S. & Goerigk, L. Effect of the damping function in dispersion corrected density functional theory. *J. Comput. Chem.* **32**, 1456–1465 (2011).
11. Shao, Y. *et al.* Advances in molecular quantum chemistry contained in the Q-Chem 4 program package. *Mol. Phys.* **113**, 184–215 (2015).
12. Weigend, F. & Ahlrichs, R. Balanced basis sets of split valence, triple zeta valence and quadruple zeta valence quality for H to Rn: Design and assessment of accuracy. *Phys. Chem. Chem. Phys.* **7**, 3297 (2005).
13. Zhang, Y., Xu, X. & Goddard, W. A. Doubly hybrid density functional for accurate descriptions of nonbond interactions, thermochemistry, and thermochemical kinetics. *Proc. Natl. Acad. Sci.* **106**, 4963–4968 (2009).
14. Su, N. Q. & Xu, X. Development of new density functional approximations. *Annu. Rev. Phys. Chem.* **68**, 155–182 (2017).
15. Chorkendorff, I. & Niemantsverdriet, J. W. *Concepts of modern catalysis and kinetics*. (Wiley-VCH Verlag GmbH & Co. KGaA, Weinheim, 2003).
16. Nørskov, J. K. *et al.* Origin of the overpotential for oxygen reduction at a fuel-cell cathode. *J. Phys. Chem. B* **108**, 17886–17892 (2004).
17. Peterson, A. A., Abild-Pedersen, F., Studt, F., Rossmeisl, J. & Nørskov, J. K. How copper catalyzes the electroreduction of carbon dioxide into hydrocarbon fuels. *Energy Environ. Sci.* **3**, 1311 (2010).
18. Letchworth-Weaver, K. & Arias, T. A. Joint density functional theory of the electrode-electrolyte interface: Application to fixed electrode potentials, interfacial capacitances, and potentials of zero charge. *Phys. Rev. B* **86**, 075140 (2012).
19. Gunceler, D., Letchworth-Weaver, K., Sundararaman, R., Schwarz, K. A. & Arias, T. A. The importance of nonlinear fluid response in joint density-functional theory studies of battery

systems. *Model. Simul. Mater. Sci. Eng.* **21**, 074005 (2013).
20. Mathew, K., Sundararaman, R., Letchworth-Weaver, K., Arias, T. A. & Hennig, R. G. Implicit solvation model for density-functional study of nanocrystal surfaces and reaction pathways. *J. Chem. Phys.* **140**, 084106 (2014).
21. Saleheen, M. & Heyden, A. Liquid-phase modeling in heterogeneous catalysis. *ACS Catal.* **8**, 2188–2194 (2018).
22. Michel, C. *et al.* Role of water in metal catalyst performance for ketone hydrogenation: a joint experimental and theoretical study on levulinic acid conversion into gamma-valerolactone. *Chem Commun* **50**, 12450–12453 (2014).
23. Liu, Y., Gregersen, B. A., Hengge, A. & York, D. M. Transesterification thio effects of phosphate desters: free energy barriers and kinetic and equilibrium isotope effects from density-functional theory. *Biochemistry* **45**, 10043–10053 (2006).
24. Kelly, C. P., Cramer, C. J. & Truhlar, D. G. Adding explicit solvent molecules to continuum solvent calculations for the calculation of aqueous acid dissociation constants. *J. Phys. Chem. A* **110**, 2493–2499 (2006).
25. Marenich, A. V., Ding, W., Cramer, C. J. & Truhlar, D. G. Resolution of a challenge for solvation modeling: calculation of dicarboxylic acid dissociation constants using mixed discrete–continuum solvation models. *J. Phys. Chem. Lett.* **3**, 1437–1442 (2012).
26. Heenen, H. H., Gauthier, J. A., Kristoffersen, H. H., Ludwig, T. & Chan, K. Solvation at metal/water interfaces: An *ab initio* molecular dynamics benchmark of common computational approaches. *J. Chem. Phys.* **152**, 144703 (2020).
27. Zhang, I. Y. & Xu, X. Gas-Phase thermodynamics as a validation of computational catalysis on surfaces: a case study of fischer-tropsch synthesis. *ChemPhysChem* **13**, 1486–1494 (2012).
28. Wang, Y., Li, Y., Chen, J., Zhang, I. Y. & Xu, X. Doubly hybrid functionals close to chemical accuracy for both finite and extended systems: implementation and test of XYG3 and XYGJ-OS. *JACS Au* **1**, 543–549 (2021).
29. Granda-Marulanda, L. P. *et al.* A Semiempirical method to detect and correct DFT-based gas-phase errors and its application in electrocatalysis. *ACS Catal.* **10**, 6900–6907 (2020).
30. Studt, F. *et al.* The mechanism of CO and CO_2 hydrogenation to methanol over Cu - based catalysts. *ChemCatChem* **7**, 1105–1111 (2015).
31. Bowker, M., Hadden, R. A., Houghton, H., Hyland, J. N. K. & Waugh, K. C. The mechanism of methanol synthesis on copper/zinc oxide/alumina catalysts. *J. Catal.* **109**, 263–273 (1988).
32. Campbell, C. T. & Daube, K. A. A surface science investigation of the water-gas shift reaction on Cu(111). *J. Catal.* **104**, 109–119 (1987).
33. Ruehl, G., Harman, S. E., Gluth, O. M., LaVoy, D. H. & Campbell, C. T. Energetics of adsorbed formate and formic acid on Cu(111) by calorimetry. *ACS Catal.* **12**, 10950–10960 (2022).
34. Muramoto, E. *et al.* Toward benchmarking theoretical computations of elementary rate constants on catalytic surfaces: formate decomposition on Au and Cu. *Chem. Sci.* **13**, 804–815 (2022).
35. Huang, Y., Handoko, A. D., Hirunsit, P. & Yeo, B. S. Electrochemical eeduction of CO_2 using copper single-crystal surfaces: effects of co* coverage on the selective formation of ethylene. *ACS Catal.* **7**, 1749–1756 (2017).
36. Calle-Vallejo, F. & Koper, M. T. M. Theoretical considerations on the electroreduction of CO

to C_2 species on Cu(100) electrodes. *Angew. Chem. Int. Ed.* **52**, 7282–7285 (2013).
37. Peng, H. *et al.* The role of atomic carbon in directing electrochemical $CO_{(2)}$ reduction to multicarbon products. *Energy Environ. Sci.* **14**, 473–482 (2021).
38. Liu, X. *et al.* pH effects on the electrochemical reduction of $CO_{(2)}$ towards C_2 products on stepped copper. *Nat. Commun.* **10**, 32 (2019).
39. NIST Chemistry WebBook https:// webbook.nist.gov/chemistry/.
40. Wellendorff, J. *et al.* A benchmark database for adsorption bond energies to transition metal surfaces and comparison to selected DFT functionals. *Surf. Sci.* **640**, 36–44 (2015).
41. Hollins, P. & Pritchard, J. Interactions of CO molecules adsorbed on Cu(111). *Surf. Sci.* **89**, 486–495 (1979).
42. Wu, K. J. & Kevan, S. D. Isothermal coverage dependent measurements of NH_3 and ND_3 desorption from Cu(001). *J. Chem. Phys.* **95**, 5355–5363 (1991).
43. Naumann d'Alnoncourt, R., Graf, B., Xia, X. & Muhler, M. The back-titration of chemisorbed atomic oxygen on copper by carbon monoxide investigated by microcalorimetry and transient kinetics. *J. Therm. Anal. Calorim.* **91**, 173–179 (2008).
44. Cao, K., Füchsel, G., Kleyn, A. W. & Juurlink, L. B. F. Hydrogen adsorption and desorption from Cu(111) and Cu(211). *Phys. Chem. Chem. Phys.* **20**, 22477–22488 (2018).
45. Díaz, C. *et al.* Chemically accurate simulation of a prototypical surface reaction: H_2 dissociation on Cu(111). *Science* **326**, 832–834 (2009).
46. King, D. A. Thermal desorption from metal surfaces: A review. *Surf. Sci.* **47**, 384–402 (1975).
47. Luo, S., Zhao, Y. & Truhlar, D. G. Improved CO adsorption energies, site preferences, and surface formation energies from a meta-generalized gradient approximation exchange–correlation functional, M06-L. *J. Phys. Chem. Lett.* **3**, 2975–2979 (2012).
48. Huber, K. P. & Herzberg, G. *Constants of diatomic molecules*. (Van Nostrand Reinhold, 1979).
49. Rempt, R. D. Electron-impact excitation of carbon monoxide near threshold in the 1.5- to 3-ev incident energy range. *Phys Rev Lett* **22**, 1034–1036 (1969).
50. Ren, X., Rinke, P. & Scheffler, M. Exploring the random phase approximation: Application to CO adsorbed on Cu(111). *Phys. Rev. B* **80**, 045402 (2009).
51. Calle-Vallejo, F., Martínez, J. I. & Rossmeisl, J. Density functional studies of functionalized graphitic materials with late transition metals for oxygen reduction reactions. *Phys. Chem. Chem. Phys.* **13**, 15639 (2011).
